# ANXA10 is a prognostic biomarker and suppressor of hepatocellular carcinoma: a bioinformatics analysis and experimental validation

**DOI:** 10.1038/s41598-023-28527-x

**Published:** 2023-01-28

**Authors:** Chaohua Zhang, Linglong Peng, Haitao Gu, Jijian Wang, Yaxu Wang, Zhiquan Xu

**Affiliations:** Department of Gastrointestinal Surgery, The Second Affiliated Hospital of Chongqing Medical University, Chongqing, 400000 China

**Keywords:** Tumour biomarkers, Data mining, Transcriptomics, Diagnostic markers, Prognostic markers

## Abstract

Liver hepatocellular carcinoma (LIHC) is one of the main cancers worldwide and has high morbidity and mortality rates. Although previous studies have shown that ANXA10 is expressed at low levels in LIHC tumor tissues, the biological function of ANXA10 in LIHC is still unclear. Therefore, we utilized TCGA, TIMER, GEPIA2, TISIDB, LinkedOmics, ssGSEA algorithms and CIBERSORT methodology to preliminarily evaluate the potential mechanism of ANXA10 in LIHC. In vitro experiments were used to further verify some functions of ANXA10. Consequently, we found that ANXA10 mRNA/protein expression was downregulated in LIHC tissue compared to normal tissue. ANXA10 was significantly linked with clinicopathological features, immunocytes, multiple cancer-related pathways, m6A modification and a ceRNA network. A three-gene prognostic signature rooted in ANXA10-related immunomodulators was determined and found to be an independent prognostic predictor. A nomogram was constructed to predict survival with good accuracy. Additionally, in vitro trials revealed that ANXA10 upregulation inhibited LIHC cell proliferation and migration. This study reveals that ANXA10 may serve as a prognostic marker and promising therapeutic target in LIHC clinical practice through various biologic functions.

## Introduction

Liver hepatocellular carcinoma (LIHC) is the fourth leading cancer-related cause of mortality globally and contributes to a high disease burden^1^. The number of LIHC cases diagnosed annually is expected to reach 1 million by 2025, presenting a serious challenge in global medicine^2^. Monitoring and early diagnosis of LIHC theoretically increase the likelihood of a cure; however, monitoring is neglected even in nations with abundant resources. Local ablation, surgical excision, or organ transplantation may be applied to cure LIHC in its early stages. Unfortunately, there are few therapeutic choices and a dismal prognosis for patients with advanced LIHC in modern management^3^. The emergence of immunotherapy, N6-methyladenosine (m6A) modification and competing endogenous RNA (ceRNA) regulatory networks has provided new therapeutic avenues for tumors. Immunotherapy has achieved remarkable clinical responses in melanoma, lung cancer, bladder cancer and so on^4^. The regulators of m6A modification have crucial roles in tumorigenesis, including proliferative, migratory, and invading functions^5^. Zhao et al. reported a newly discovered ceRNA network that may provide significant research resources for the treatment of colorectal cancer^6^. Therefore, we sought identify new biomarkers and explore their potential roles in the immune response, m6A modification and ceRNA network in LIHC.

Annexin A10 (ANXA10), an annexin family member, is a protein-coding gene located on chromosome 4q33^7^. Annexins comprise the calcium-binding family of proteins that bind to membranes. They have numerous biological activities, including apoptosis, vesicle trafficking, calcium signaling, growth control and cell division^8^. A previous study reported that ANXA10 is a malignancy inhibitor, and knockdown of ANXA10 can stimulate lung cancer cells to invade and metastasize^9^. In addition, absence of ANXA10 expression is closely linked with a worse prognosis in early-stage gastric cancer^10^. Although it has been previously confirmed that decreased expression of ANXA10 correlates with a poor prognosis for liver cancer^11^, there is still no literature about the biomedical mechanism and function of ANXA10 in LIHC.

In our research, we analyzed the differential expression of ANXA10 in LIHC. From multidimensional analysis, we carried out enrichment analysis of coexpressed genes, searched for the relationships between genes and immunity, and developed a predictive model of prognosis. We also explored the relationships between ANXA10 and m6A modification and the ceRNA network. Finally, we experimentally verified some of the predicted results.

## Methods

### TCGA data

The Cancer Genome Atlas (TCGA) is a free public database that includes more than 10,000 samples across 39 tumor types^12^. We obtained data on LIHC from the TCGA website (https://portal.gdc.cancer.gov/). The data included transcriptome data and clinicopathological data, which were processed by R software^13^. Transcriptome data and mRNA sequencing data (HTSeq-FPKM) of 374 tumor tissues and 50 normal tissues were included. Gene expression data normalized to transcripts per million (TPM) were converted to log2(TPM + 1) form. Clinicopathological data of 377 cases were included. In this current research, we analyzed the difference in ANXA10 expression between tissue types and its connection with clinicopathological features using TCGA data. Additionally, the R package GSVA was implemented to analyze the LIHC-TCGA data to calculate the immune cell infiltrating with the ssGSEA algorithm. ssGSEA uses the specific markers of each type of immune cell as a gene set to compute the enrichment score of each type of immunocyte in every specimen to reflect the infiltration of immunocytes^14^. Additionally, the CIBERSORTx method was also utilized to appraise the enrichment of some immunocytes. CIBERSORTx (https://cibersortx.stanford.edu/) is the successor to CIBERSORT and is used for bulk RNA-Seq data deconvolution^15^. To determine the disparities in m6A-related gene expression, LIHC-TCGA samples were divided into two groups based on the median value of ANXA10 expression (low and high expression groups). In accordance with a previous literature report^16^, m6A-related genes included writers (WTAP, VIRMA, RBM15B, RBM15, METTL3, METTL14, ZC3H13), erasers (ALKBH5, FTO) and readers (RBMX, HNRNPC, YTHDC2, YTHDC1, IGF2BP3, IGF2BP1, IGF2BP2, HNRNPA2B1, YTHDF3, YTHDF1, YTHDF2).

### GEO data

GEO is a public functional genomics data repository supporting MIAME-compliant data submissions^17^. GSE54236 and GSE14520 datasets were downloaded from https://www.ncbi.nlm.nih.gov/geo/. The GEO data was also processed by R software. The GSE14520 data was used to explore differential gene expression, survival analysis and validation of prognosis signature. The GSE54236 was conducted to determine the disparities in m6A-related gene expression according to ANXA10 expression (low and high expression groups).

### TIMER

Tumor IMmune Estimation Resource (TIMER, https://cistrome.shinyapps.io/timer/) is a useful guide for scientific evaluation of immunologic infiltration among multiple cancer types^18^. In the current research, the “DiffExp” module was utilized for assessing the expression differences of ANXA10 in pan-cancer. Besides, the “SCNA” module was conducted to evaluate the relation between immune cells and the somatic copy number alteration (SCNA).

### GEPIA2

Gene Expression Profiling Interactive Analysis 2 (GEPIA2) (http://gepia2.cancer-pku.cn/#index) is an upgraded version that performs gene expression studies primarily on cancerous and normal samples through the GTEx and TCGA databases^19^. In our research, the “box plot” and “stage plot” modules of GEPIA2 were used to conduct expression difference research and contrast ANXA10 expression in various pathological stages separately.

### LinkedOmics analysis

The LinkedOmics database (http://www.linkedomics.org/login.php) comprises clinical and multiomics data for 32 kinds of cancer and 11,158 individuals from the TCGA program^20^. The Spearman correlation test was employed to identify genes with a significant connection. The “LinkFinder” module displayed the result of the association analysis as a table, heatmap, and volcano graph. The “LinkInterpreter” module can execute gene set enrichment analysis (GSEA), including Gene Ontology (GO) and Kyoto Encyclopedia of Genes and Genomes (KEGG) pathways, the former of which contains cellular component (CC), biological process (BP) and molecular function (MF). The criteria for selection were 500 simulations and p < 0.05.

### TISIDB

TISIDB (http://cis.hku.hk/TISIDB/) is an intuitive web gateway that integrates many types of research data in oncoimmunology^21^. We explored whether there was a link between ANXA10 and the grade of LIHC patients through the “Clinical” module. The “Lymphocyte” module was applied to determine the effect of the expression and copy number (CNA) of ANXA10 on the content of tumor-infiltrating lymphocytes (TILs). Subsequently, the “Immunomodulator” module was used to study the relation between immunomodulators and ANXA10 expression. We selected immunostimulators and immunoinhibitors whose gene expression was strongly linked with that of ANXA10 in LIHC (Spearman test, p < 0.05). Subsequently, we performed functional enrichment analysis of relevant immunomodulators through WebGestalt (http://www.webgestalt.org/)^22^.

### Establishment and evaluation of the prognostic signature

We aimed to build a predictive multiple immunological gene signature using immunomodulators connected with ANXA10. Stepwise variable selection was carried out utilizing the Akaike information criterion in Cox models^23^. After the related immunomodulators were settled, a model for the risk score—a prognostic index—was generated: risk score = β1X1 + β2X2 + ⋯ + βiXi, where βi and Xi represent the risk coefficient and expression extent of the related immunomodulators originating from the Cox model, respectively^24^. The correlation of clinical characteristics and prognostic signature score with overall survival were assessed by log-rank test, univariate Cox analyses and Kaplan–Meier survival curve analysis. Multivariate analysis was conducted to evaluate the ability of the prognostic index to predict survival after adjusting for sex, age, stage and grade. The predictive precision of the prognostic index was assessed by the time-dependent receiver operating characteristic (ROC) curve with the survival ROC package^25^.

### Construction and validation of the nomogram

Nomograms are becoming increasingly common in cancer prognosis prediction. To account for the profile of an individual patient, nomograms can transform statistical predicting models into numerical assessments of probabilities of individual events, for example, recurrence or death^26^. In this study, we utilized clinical characteristics and the prognostic index to design a nomogram based on the rms R package. We plot calibration curves to convey the discrepancy of the forecasted probability and the actual probability of occurrence.

### Construction of the ceRNA network

To obtain ANXA10-related ceRNA networks, we first used starBase 3.0 (http://starbase.sysu.edu.cn/) for forecasting the targeted microRNAs (miRNAs) of ANXA10, and the predicted results included two databases, miRanda and PITA^27^. TargetScan was also employed to predict ANXA10-targeted miRNAs^28^. In addition, the association between ANXA10 expression and targeted miRNA expression was analyzed to identify miRNAs that correlated more closely with ceRNA settings. Next, we used starBase and miRNet2.0 (www.mirnet.ca/miRNet/home.xhtml) to find miRNA-targeting long noncoding RNAs (lncRNAs). Similarly, we examined the link between targeted miRNAs and lncRNAs at the expression level to identify lncRNAs that were more suitable with ceRNA settings. Systematic investigation of mRNA–miRNA and miRNA–lncRNA pairs with inverse expression trends was conducted to construct a crucial lncRNA–miRNA-mRNA (ANXA10) ceRNA network.

### Immunohistochemistry

This study was approved by the Ethics Committee of the Second Affiliated Hospital of Chongqing Medical University and acquired informed consent from all patients who participated in this study. All methods were performed in accordance with the Helsinki declaration guidelines and regulations. The tissue samples of 20 patients with LIHC were obtained from the Second Affiliated Hospital of Chongqing Medical University. Recombinant anti-Annexin A10 was purchased from Abcam. Tissues embeded in paraffin were sliced into 3 µm thick sections. Antigen was repaired by sodium citrate solution (Beyotime, China). Immunohistochemistry (IHC) was carried out with the detection system of polymer horseradish peroxidase (Zhongshan Goldenbridge Biotechnology, China). The incubation of sections is the following specific primary antibodies: Anxa10(Abcam, ab213656, 1:1000).

### Quantitative real-time PCR (qRT-PCR)

Total RNA was isolated from cells with TRIzol (TaKaRa). Genomic DNA was removed from the RNA, and the PrimeScript RT Reagent kit was applied for reverse transcription into cDNA (TaKaRa). RNA expression was measured by qRT‒PCR using TB Green Premix Ex Taq II (TaKaRa). β-Actin was used as a normalizing control for mRNA. In our research, the following primer sequences were used: human Anxa10, 5′-ATG ATT GCA GAG GCA TAC CAG A-3′ (forward) and 5′-GCC AGC CAT CAC ATC TTT GAA-3′ (reverse); human β-actin, 5′-CCT TCC TGG GCA TGG AGT CCT-3′ (forward) and 5′-GGA GCA ATG ATC TTG ATC TT-3′ (reverse).

### Culture and transfection

The Institute of Biochemistry and Cell Biology provided a normal liver cell line (LO2) and two LIHC cell lines (LM3 and Huh7) (Chinese Academy of Sciences, Shanghai, China). All cell lines were cultivated in Dulbecco's modified Eagle’s medium (Gibco, USA) supplemented with 100 units per ml of streptomycin and penicillin (HyClone, USA) and 10% fetal bovine serum (FBS, Gibco, USA). The humidified incubator containing 5% CO2 was applied to nurture cells at 37 °C. Cells were transfected with the Anxa10 plasmid or its negative control (NC) plasmid by Lipofectamine 2000(Invitrogen) according to the product’s guidelines. The Anxa10 and NC plasmids were obtained from Genechem (Shanghai, China).

### Western blotting (WB)

Total protein was extractedwith RIPA lysis buffer (Beyotime, China). The samples were incubated on ice for 15 min, and the supernatant was collected after centrifugation at 12,000 r/min at 4 °C for 10 min. The protein concentration was determined using a bicinchoninic acid kit (Beyotime, China) in conformity with manufacturer’s instructions. After adding loading buffer (Beyotime, China), protein was separated by sodium dodecyl sulfate-polyacrylamide gel electrophoresis, and protein was transferred to a nitrocellulose transfer membrane (GE Healthcare, USA). The membranes were blocked for one hour at room temperature with 5% nonfat powdered milk, and the blots were probed overnight at 4 °C with the following specific primary antibodies: Anxa10 (Abcam, ab213656, 1:1000) and β-actin (Proteintech, 20536-1-AP, 1:1000). Tris-buffered saline Tween-20 (TBST) was applied to wash the membrane 3 times, and the incubation of membrane was anti-rabbit immunoglobulin G(IgG) (CST, 7074, 1:5000) secondary antibody at room temperature for 1 h. After washing, the protein bands were observed using Bio-Rad Image Analysis System (BIO-RAD, USA) with an improved chemiluminescence Fluorescence Detection kit (GE Healthcare, USA).

### MTT assay

The MTT (Beyotime, China) assay was utilized to observe cell proliferation. Huh7 and LM3 cells were seeded at 3000 cells/well in 96-well plates. 10 μl of MTT reagent (5 mg/ml) was added into transfected wells and cultured at 37 °C for 4 h. After removing the growth medium, 100 µl DMSO was applied to each well to dissolve the formazan. Absorbance at 490 nm was detected to assess cell proliferation by using an enzyme calibration system (ThermoFisher).

### Colony formation assay

Huh7 and LM3 cells were seeded in 6-well plates (500 cells per well) comprising 2 ml of culture medium and then cultivated for 2–3 weeks to generate monoclonal cells. The concentration of formaldehyde fixation of colonies was 4% and the staining of colonies was 0.1% crystal violet for 15 and 30 min, separately. The colonies were counted to assess cell proliferation.

### 5-Ethynyl-2′-deoxyuridine (EdU) assay

The 5-ethynyl-2′-deoxyuridine (EdU) (Beyotime, China) assay was conducted to assess cell proliferation accordingto the manufacturer’s manual. In shortly, 5 × 10^4^ Huh7 and LM3 cells were grownin 24-well plates. After 24 h, the cells were preheated with EdU (10 μM) at 37 °C for 2 h and fixed with 4% formaldehyde for 15 min. After washing with phosphate buffered saline (PBS) 3 times, 0.3% Triton X-100 solution was used to permeabilize the cells for 10 min at room temperature. After washing again, the click additive solution proceeded for 0.5 h in the dark room. To measure the percentage of cell proliferation, cells were stained by adding 1× Hoechst 33342 at room temperature for 10 min in the dark. Typical images were photographed under an inverted microscope, and the rate of EdU-positive cells was measured by ImageJ.

### Transwell assay

Transwell assays were performed to assess cell migration using 24-well transwell chamber. The lower chamber was filled with 500 µl DMEM comprising 10% FBS, and 100 µl DMEM containing a cell suspension was added to the upper chamber at a concentration of 1 × 106 cells/ml. After 24 h, the Transwell chambers were removed, fixed and stained with 4% formaldehyde and 0.1% crystal violet for 15 and 10 min, respectively. The cells were counted and photographed under an inverted microscope.

### Statistical analysis

R software version 4.0.5 was used to perform the statistical analysis. Clinical factors related with overall survival (OS) were evaluated by multivariate and univariate Cox regression analyses. Experimental data were analyzed by GraphPad Prism 8 with a T-test. Unless otherwise stated, p < 0.05 represented statistical significance.

## Results

### Differential expression of ANXA10 in LIHC

**Figure 1:**
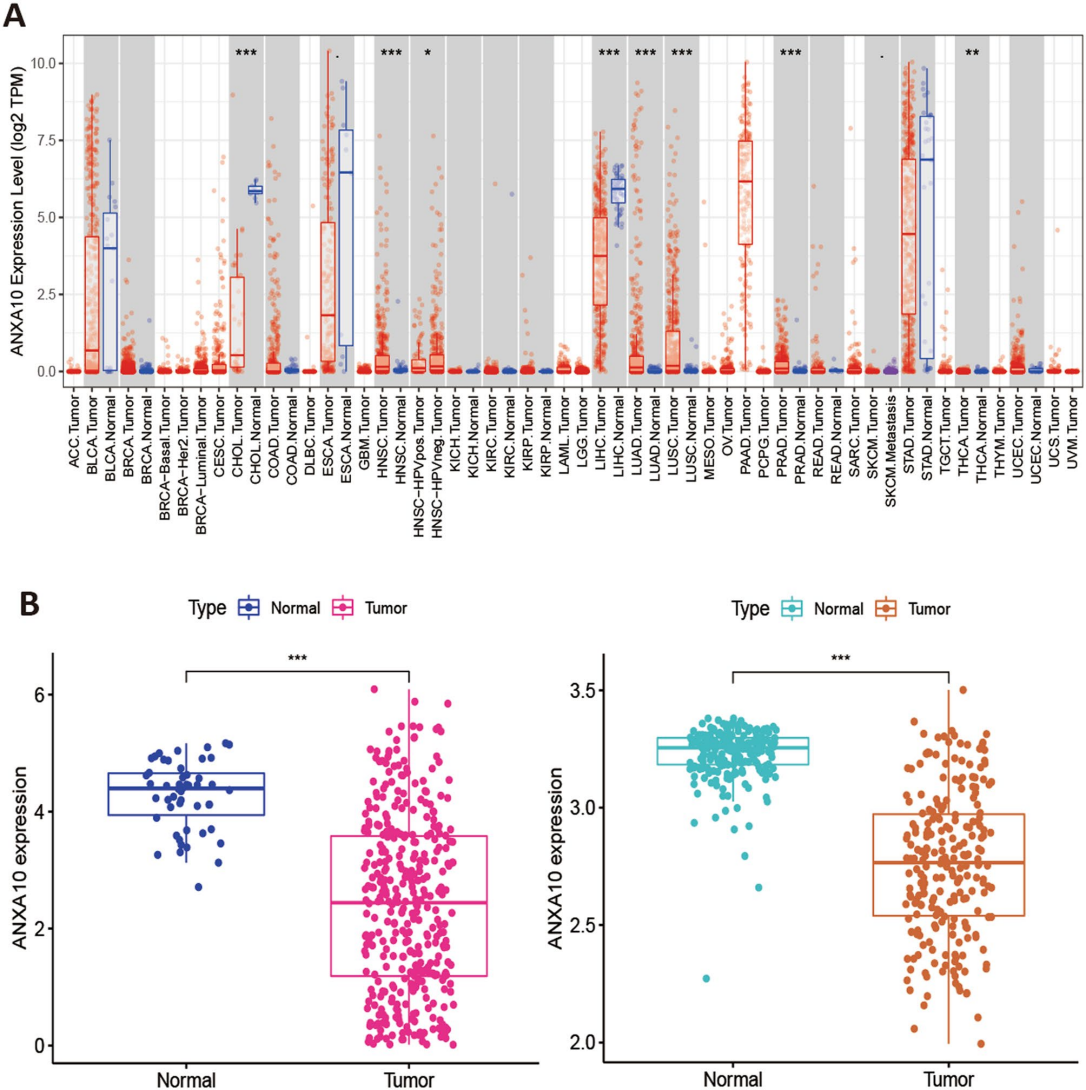

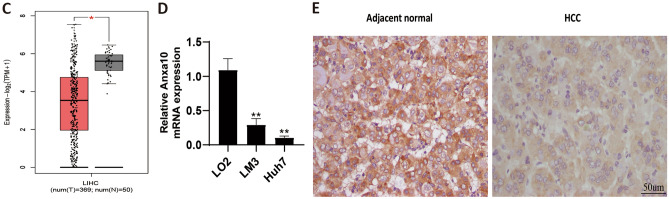
ANXA10 expression in LIHC. (**A**) Expression differences across cancers from TIMER. (**B, C**) ANXA10 differential expression analysis was performed using TCGA (**B left**), GSE14520 (**B right**) and GEPIA2 databases. (**D**) Differential expression of ANXA10 in LIHC cell lines and a liver cell line. (**E**) ANXA10 protein expression in LIHC and adjacent normal tissues by IHC. *p < 0.05; **p < 0.01; ***p < 0.001.

TIMER, R software and GEPIA2 were used to determine the difference in ANXA10 at the mRNA level, Fig. 1A shows that compared to normal adjacent tissues, expression of ANXA10 was significantly increased in lung squamous cell carcinoma (LUSC), prostate adenocarcinoma (PRAD), head and neck squamous cell carcinoma (HNSC), lung adenocarcinoma (LUAD) and thyroid carcinoma (THCA) but decreased in cholangiocarcinoma (CHOL) and liver hepatocellular carcinoma (LIHC). The results of R software analysis (Fig. 1B) and GEPIA2 (Fig. 1C) revealed that ANXA10 was abundantly expressed in normal tissues in contrast to LIHC. To confirm the database analysis results, qRT–PCR and an immunohistochemistry (IHC) were conducted. qRT–PCR showed that ANXA10 expression was considerably lower in human LIHC cell lines (LM3 and Huh7) than in a normal human liver cell line (LO2) (Fig. 1D). The IHC results (Fig. 1E) proved that ANXA10 protein expression was downregulated in tumor tissues.

### Clinicopathological analysis

**Figure 2:**
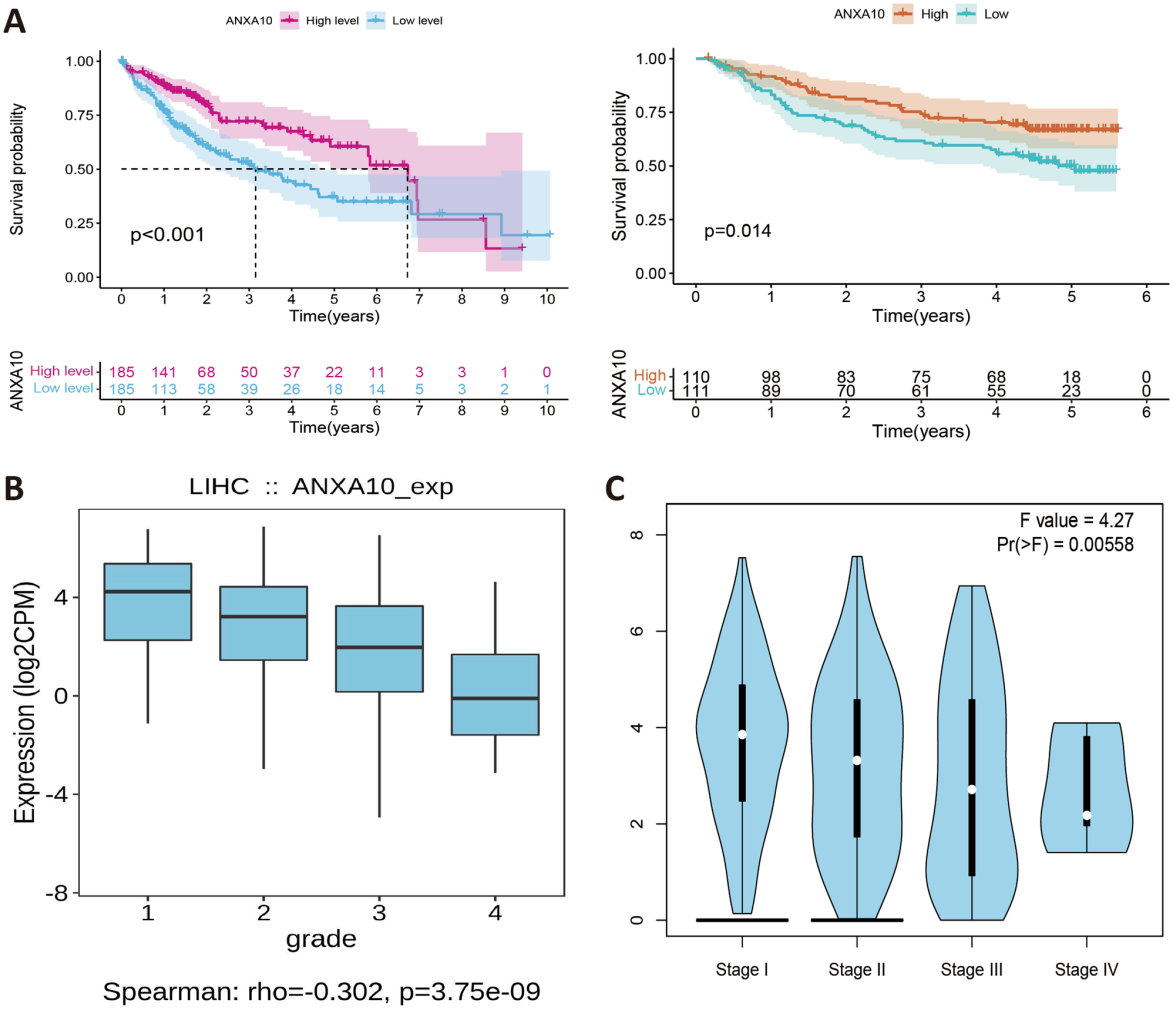
Correlations between ANXA10 and clinicopathological traits. (**A**) Survival analysis of ANXA10 in TCGA-LIHC (left) and GSE14520 (right). (**B,C**) Association of ANXA10 expression with LIHC grade and stage from TISIDB and GEPIA2, respectively.

To further analyze the relationship between ANXA10 and clinicopathological factors, R software, TISIDB and GEPIA2 were used. Figure 2A showed that ANXA10 overexpression was considerably correlated with better OS (p < 0.001) with TCGA data and GSE14520 data. In addition, the results of TISIDB (Fig. 2B) and GEPIA2 (Fig. 2C) revealed that the grade and stage of LIHC gradually decreased with increasing ANXA10 expression. These findings indicate that ANXA10 might play an active role in LIHC.

### Enrichment analysis of genes coexpressed with ANXA10

**Figure 3:**
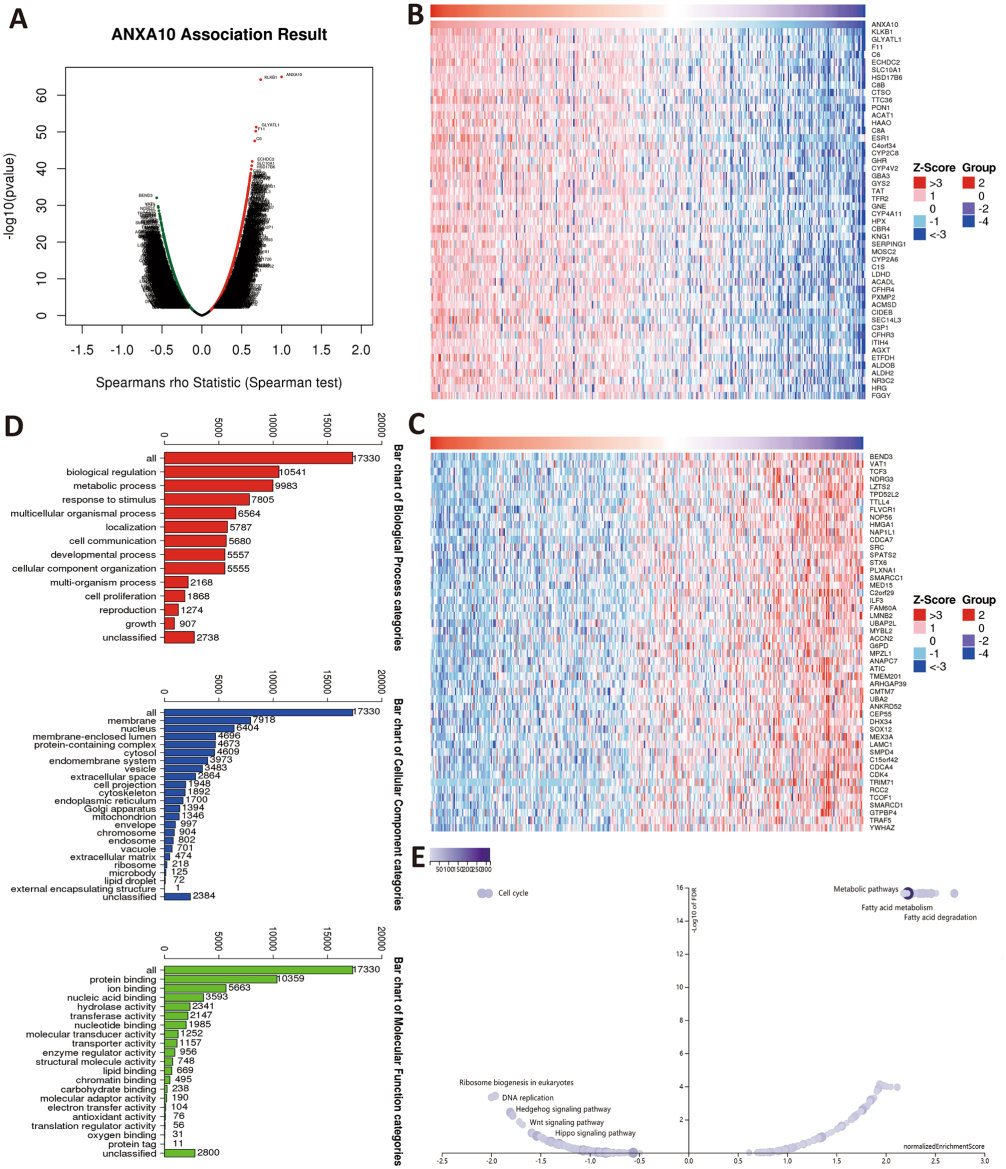
Genes coexpressed with ANXA10 and enrichment analyses by LinkedOmics. (**A**) ANXA10-related coexpressed genes are displayed in a volcano plot. (**B,C**) Heatmaps depict the top 50 coexpressed genes positively and negatively correlated with ANXA10 expression in LIHC. (**D,E**) The GO and KEGG analyses of coexpressed genes were downloaded from LinkInterpreter.

To further comprehend the underlying mechanisms of ANXA10 in LIHC, we explored ANXA10 coexpressed genes and performed enrichment analysis through the LinkedOmics database. As depicted in Fig. 3A, ANXA10 expression was positively associated with 7727 genes and negatively correlated with 12195 genes (p < 0.05). Figure 3B,C presented the top 50 genes with positive and negative correlations with ANXA10, respectively. Moreover, “LinkInterpreter” was utilized for further GO and KEGG enrichment analyses. GO function annotation (Fig. 3D) showed that ANXA10 was mainly involved in biological regulation, metabolic processes, nucleic acid binding, ion binding, and protein binding and was localizes to the cell membrane. KEGG pathway analysis (Fig. 3E) showed that ANXA10 was mainly related to Cell cycle, Ribosome biogenesis in eukaryotes, DNA replication, Wnt signaling pathway, Hippo signaling pathway, Metabolic pathways, Fatty acid metabolism, Fatty acid degradation and Hedgehog signaling pathway.

### Relationship between ANXA10 and tumor immune infiltrates

**Figure 4:**
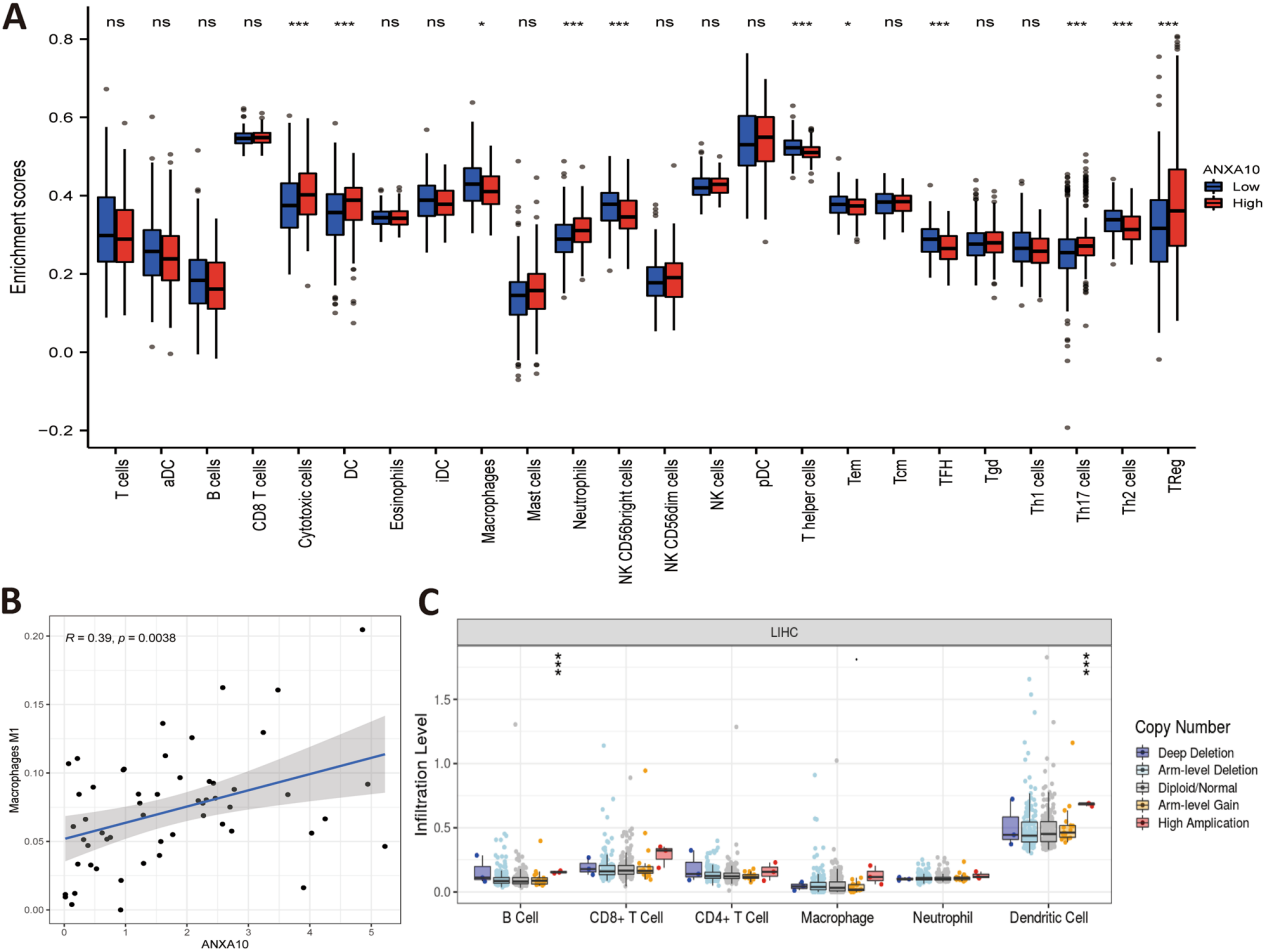
Relationships between ANXA10 and the abundance of immune cells in LIHC. (**A**) Assessment of the correlation between ANXA10 and the levels of immune cells by ssGSEA algorithm. (**B**) Scatter diagram showing the connection between ANXA10 and M1 macrophages by CIBERSORTx. (**C**) Comparative analysis of tumor infiltration degree based on ANXA10 SCNA. *p < 0.05; **p < 0.01; ***p < 0.001.

**Figure 5:**
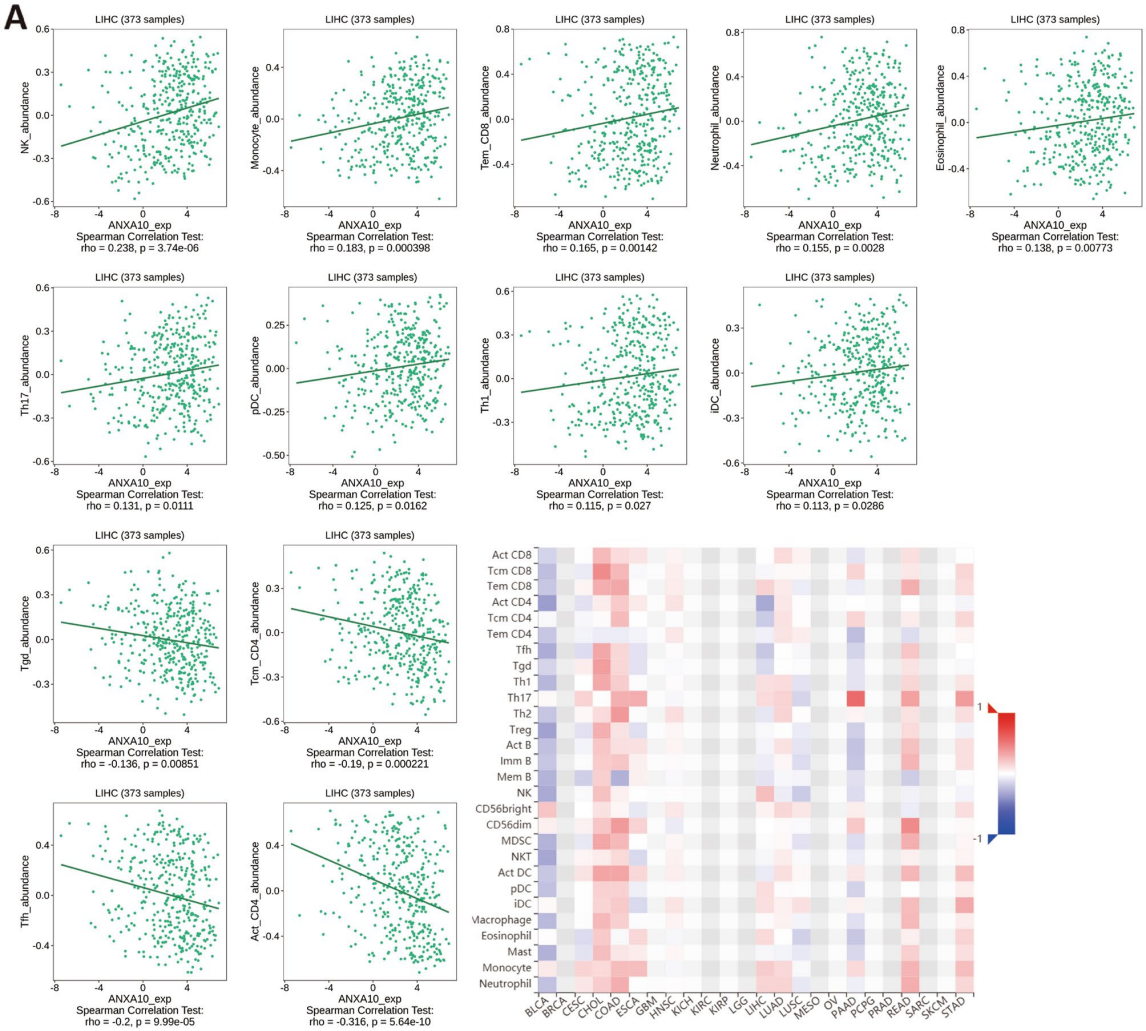

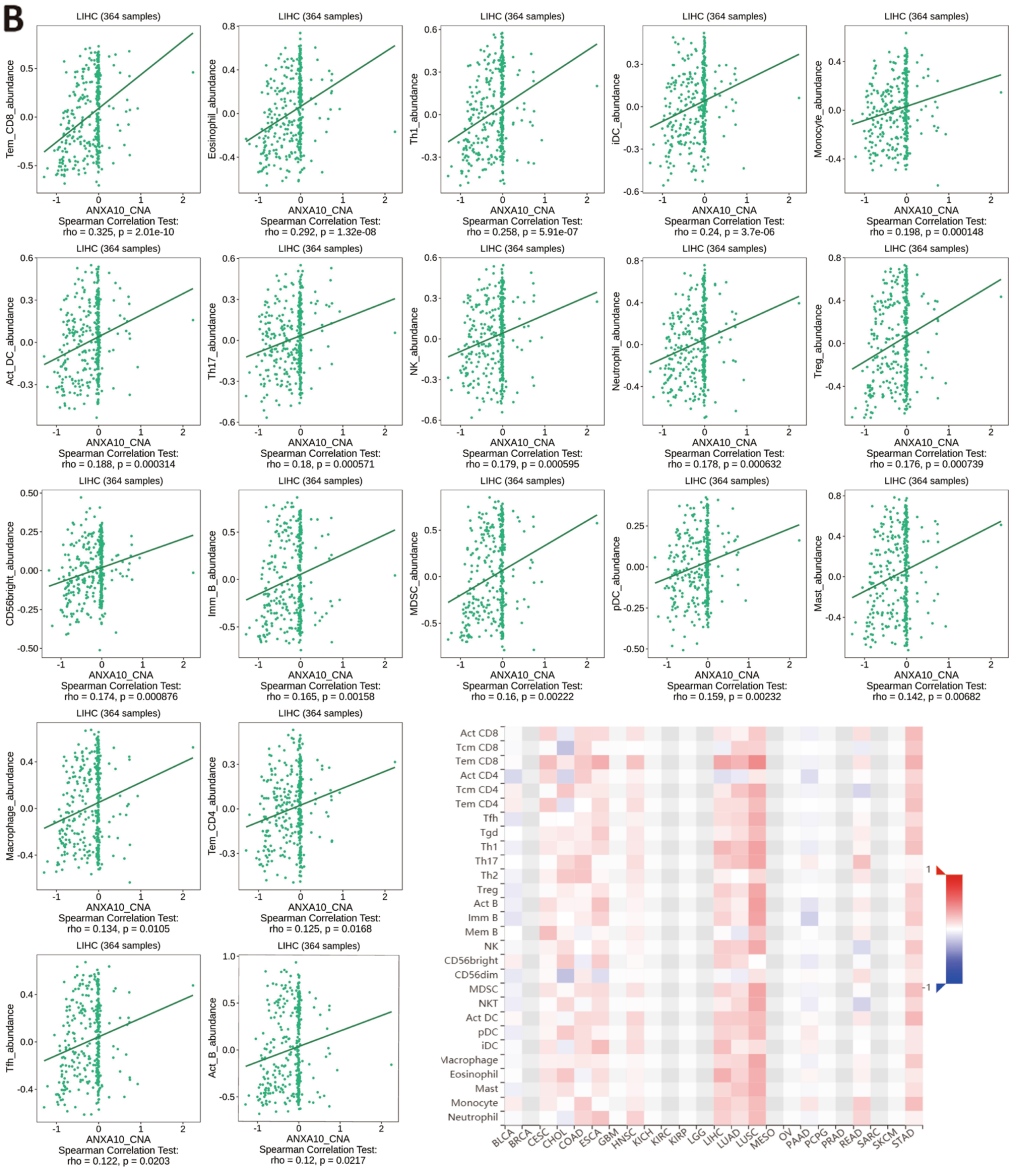
Relevance of the abundance of TILs and ANXA10 in LIHC. (**A**) Relation between the abundance of TILs and ANXA10 expression. (**B**) Relevance of the abundance of TILs and ANXA10 copy number.

Hepatocellular carcinoma (HCC) occurs as a regular inflammation-related tumor, and immune evasion is among the characteristics of HCC occurrence and evolution^29^. The extent of immunosuppression in the tumor microenvironment (TME) is intimately connected with the poor prognosis of LIHC patients^30^. To further investigate whether ANXA10 plays an immune role in LIHC, we performed multidimensional analysis with different tools. The ssGSEA results identified variations in 24 immune cell types in the low ANXA10 expression and high ANXA10 expression groups in LIHC. Figure 4A showed that the proportions of Cytotoxic cells, DC, Macrophages, Neutrophils, NK CD56bright cells, T helper cells, Tem, TFH, Th17 cells, Th2 cells and TReg were significantly related to ANXA10 expression. Among them, there were greater ratios of Cytotoxic cells, DC, Neutrophils and Th17 cells in the high-expression group. In contrast, the proportions of Macrophages and NK CD56bright cells were lower in the high expression group. Similar to some immune cells, such as macrophage, which have been reported to occur in both anti-tumor and pro-tumor forms^31^, so we utilized CIBERSORTx for further investigation. The result of CIBERSORTx (Fig. 4B) showed that M1 Macrophages was positively correlated to ANXA10 expression. Additionally, the TIMER SCNA module displayed that the copy number of high amplication was positively correlated with Dendritic and B cell (Fig. 4C). At the same time, the results in Fig. 4A showed that ANXA10 was associated with lymphocytes. Therefore, TISIDB’s “Lymphocyte” module was used to explore the relations between abundance of tumor-infiltrating lymphocytes (TILs) and ANXA10 expression. In LIHC, the abundance of TILs was either negatively or positively related to ANXA10 expression (Fig. 5A) and positively related to ANXA10 copy number (Fig. 5B), respectively.

**Figure 6:**
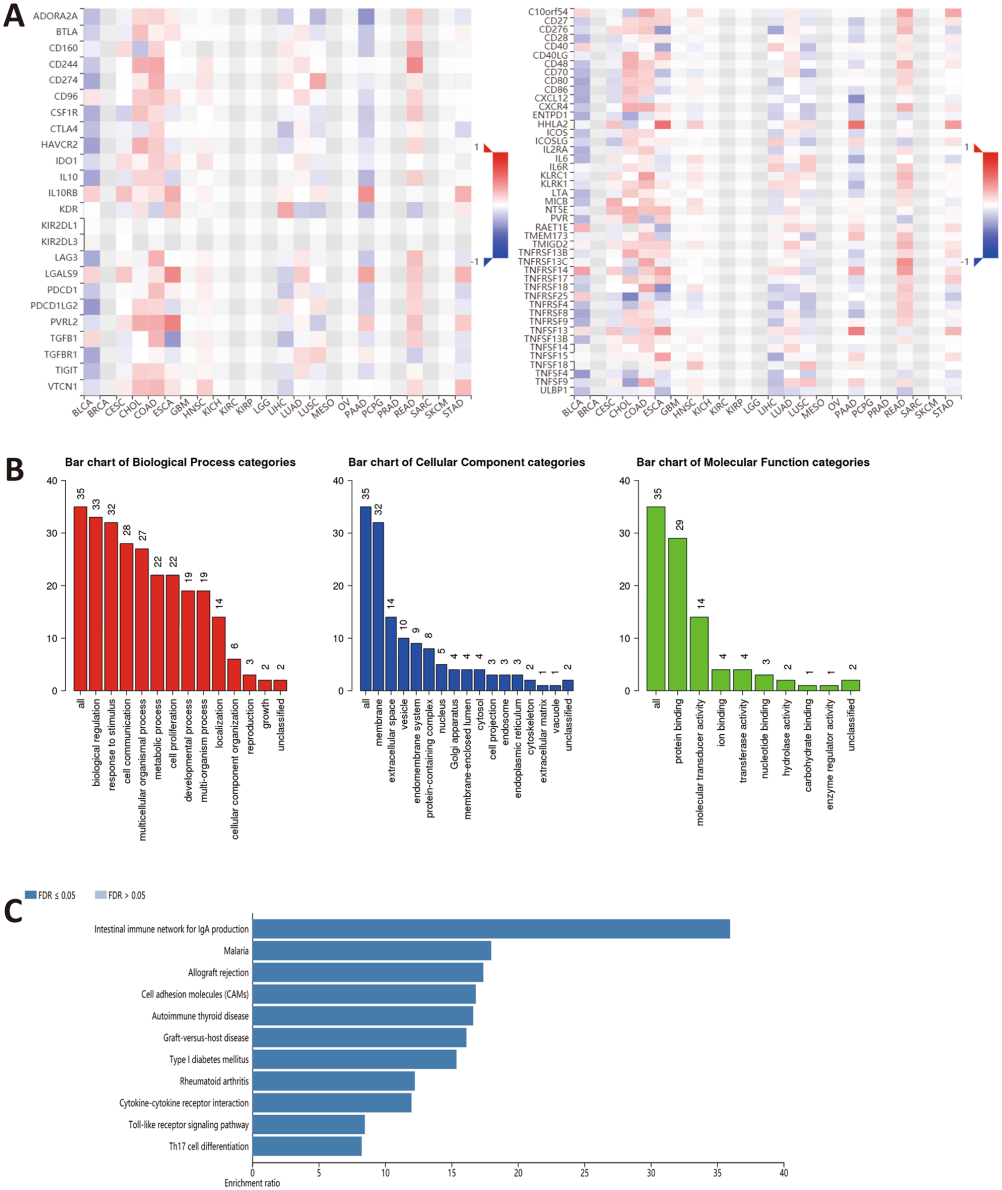
Correlation and analysis of immunomodulators related to ANXA10. (**A**) Correlation heatmaps showed that ANXA10 expression was associated with immunoinhibitors (left) and immunostimulators (right). (**B,C**) GO and KEGG analyses of ANXA10-related immunomodulatory genes via WebGestalt.

To further determine the potential immunomodulatory function of ANXA10 in LIHC, TISIDB and WebGestalt were used to conduct a series of investigations. Based on TISIDB, we identified 25 immunostimulators (CD40, ICOS, ENTPD1, CXCR4, CD276, CD86, CD80, ICOSLG, TNFRSF4, NT5E, MICB, LTA, KLRK1, IL6R, IL6, IL2RA, TNFRSF8, TNFRSF18, TNFRSF9, TNFRSF14, TNFRSF25, ULBP1, TNFSF15, TNFSF9, TNFSF4) and 10 immunoinhibitors (ADORA2A, CTLA4, HAVCR2, PDCD1, LGALS9, KDR, TGFB1, PDCD1LG2, TIGIT, VTCN1), all of which were statistically significant (Fig. 6A). Subsequently, ANXA10-related immunomodulators were subjected to enrichment analysis by WebGestalt. GO and KEGG analysis of these genes suggest that Cytokine-cytokine receptor interaction, Toll-like receptor signaling pathway, Cell adhesion molecules and Th17 cell differerntiation are related to ANXA10-induced immune events (Fig. 6B,C).

### Construction of gene prognostic signature

**Figure 7:**
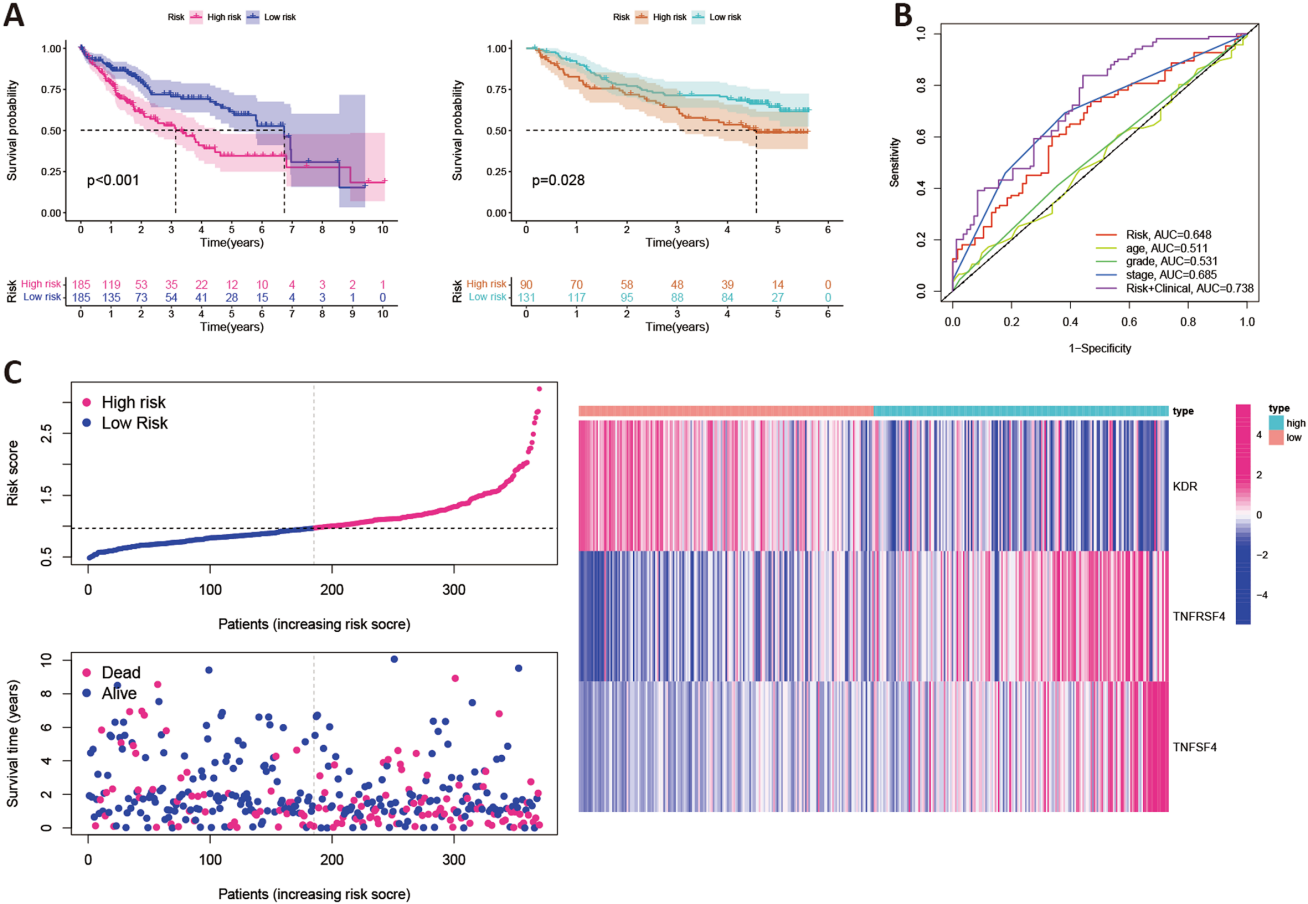

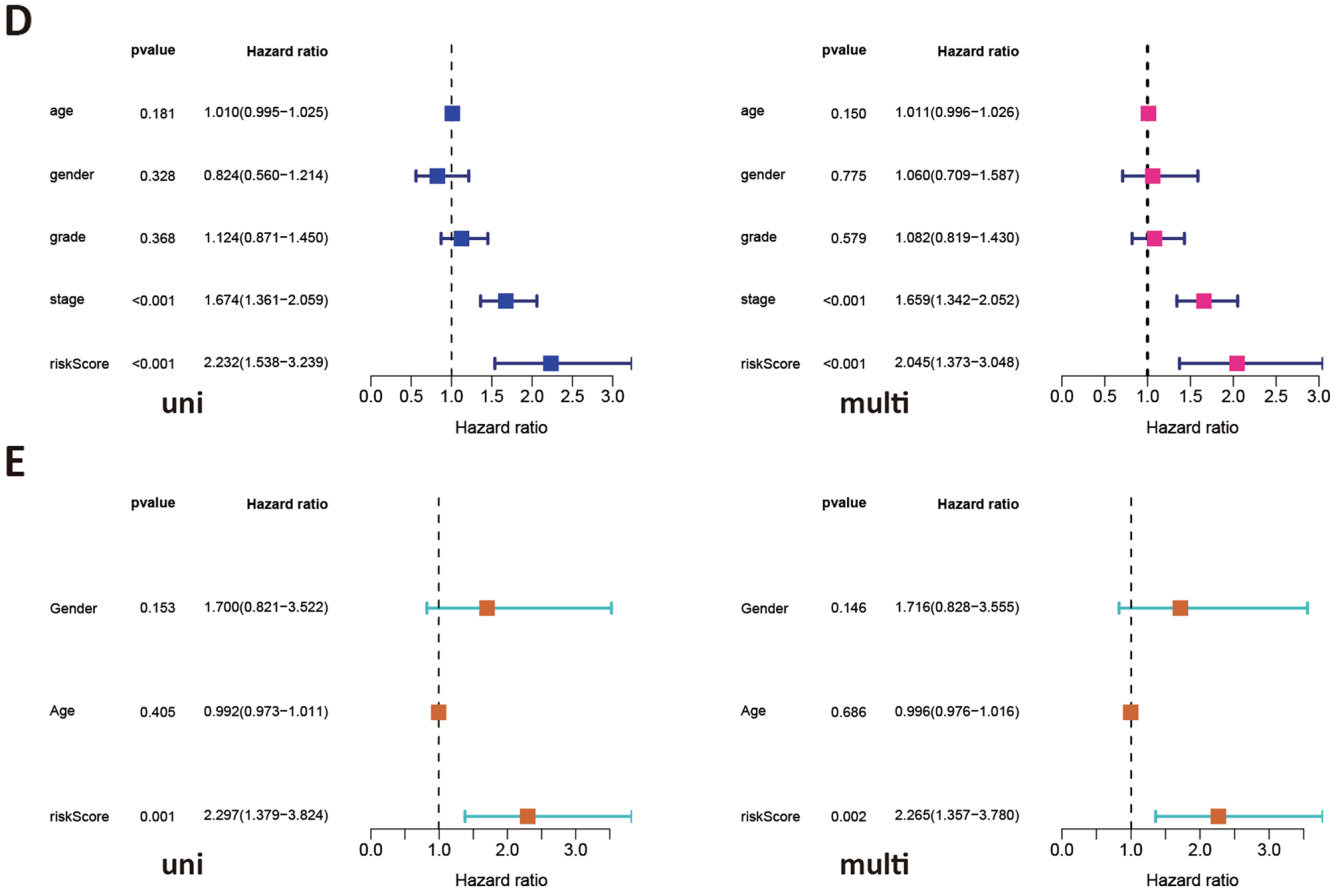
The prognostic values of the risk score originated from TCGA database. (**A**) Kaplan–Meier curve with respect to the risk score in TCGA (left) and GSE14520 (right) data. (**B**) Time-dependent ROC curves at 3-years for LIHC. (**C**) The landscape of risk score, survival status, and genes expression profiling in TCGA-LIHC. Univariate and multivariate Cox regression analyses of the risk score in LIHC with TCGA (**D**) and GSE14520 data (**E**).

To determine the predictive utility of ANXA10-associated immunomodulators in TCGA-LIHC, these immunomodulators were subjected to multivariable stepwise Cox regression. Three prognostic immunomodulators were identified and used to build a 3-gene prognostic model. Table 1 displayed the biological function of the three genes and their risk coefficients. The risk score of TCGA-LIHC patients was then calculated by the previously mentioned formula. The median risk score (0.97) divided TCGA-LIHC patients into a high-risk group (185 cases) and a low-risk group (185 cases). To increase the reliability of the prognostic model, we also use GSE14520 data to calculate risk scores. The Kaplan–Meier plot demonstrated that low-risk individuals had considerably longer OS periods than high-risk individuals (p < 0.001, Fig. 7A) in TCGA data and GEO data. ROC curves were used to assess the prognostic accuracy of the gene signature. As shown in Fig. 7B, the area under the curve (AUC) values of the risk score, age, grade and stage were 0.648, 0.511, 0.531 and 0.685, respectively. When age, grade, stage and risk score were combined, an AUC of 0.738 was achieved. Figure 7C revealed the landscape of risk score, survival status, and genes expression profiling in the TCGA-LIHC patients. Additionally, univariable and multivariable Cox regression models were conducted to further appraise the 3-gene prognostic model (Fig. 7D). Univariable Cox regression revealed that the risk score was strongly associated with OS (HR 2.232, 95% CI 1.538 − 3.239, p < 0.001). After adjusting for age, sex, grade and stage, multivariable Cox regression demonstrated that the risk score was an independence predictor of prognosis in LIHC (HR 2.045, 95% CI 1.373 − 3.048, p < 0.001). Additionally, in GSE14520 data, Fig. 7E showed that the risk score was still an independence predictor of prognosis after the univariable and multivariable Cox regression.

**Table 1:** Function of the genes in the prognostic signature.

Gene symbol	Function	Risk coefficient	p value
KDR	Tyrosine-protein kinase that acts as a cell-surface receptor for VEGFA, VEGFC and VEGFD	− 0.2164923	0.0342
TNFRSF4	A costimulatory molecule implicated in long-term T-cell immunity	0.2451178	0.0399
TNFSF4	Co-stimulates T-cell proliferation and cytokine production	0.3069508	0.0158

### Establishment and evaluation of nomogram

**Figure 8:**
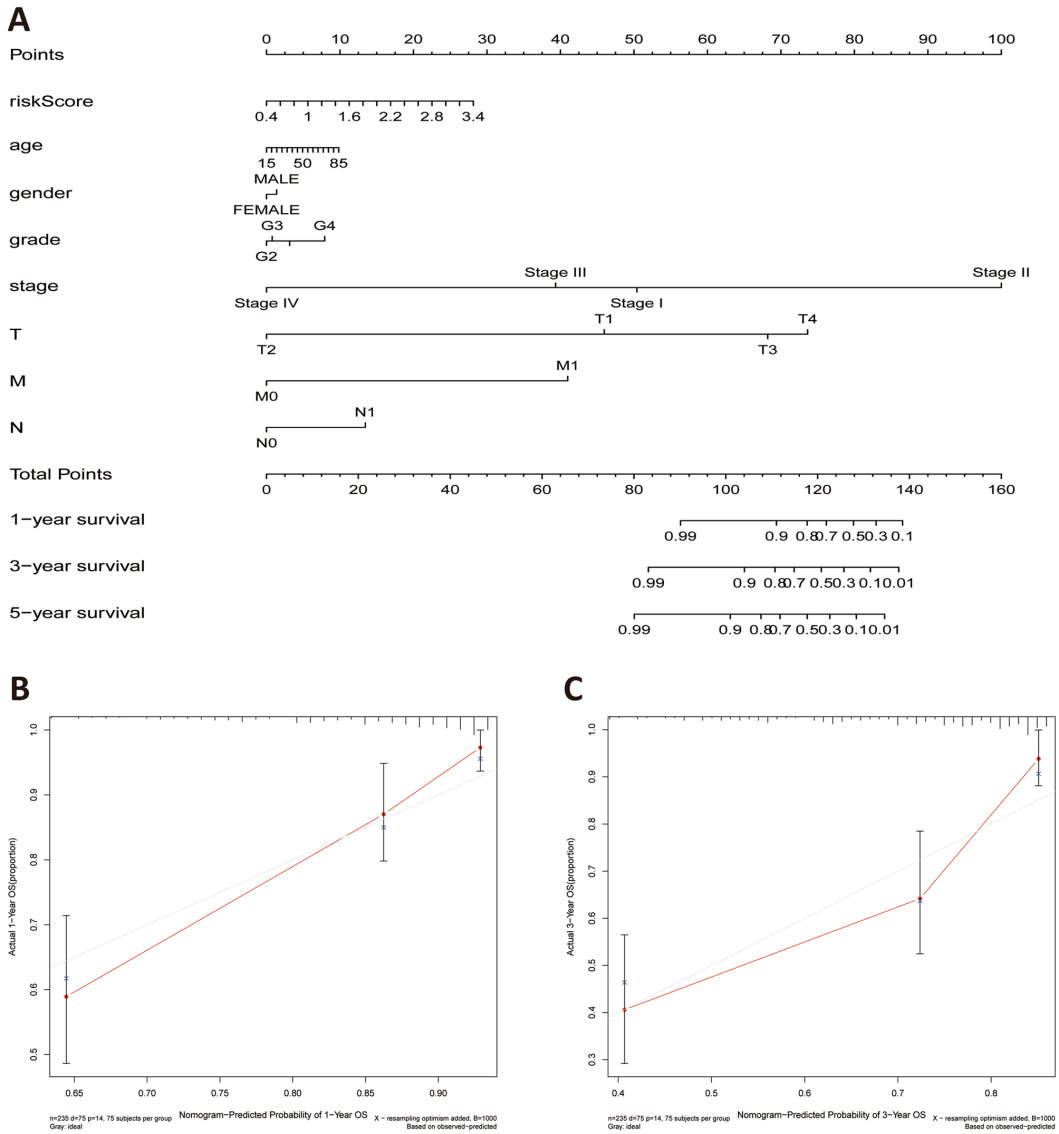

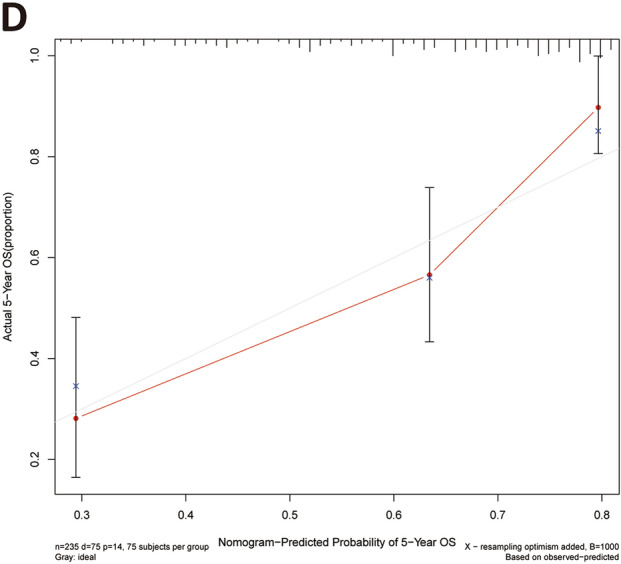
Generation of the prognostic nomogram using TCGA data. (**A**) Nomogram for forecasting the 1-, 3- and 5-year OS in LIHC patients. (**B–D**) OS calibration curves for LIHC patients at 1, 3, and 5 years. The X and Y axes define the nomogram-predicted OS and the actual OS, respectively.

To better elucidate the potential clinical validity of the TCGA-LIHC risk model, a prognostic nomogram was established generated from the TCGA-LIHC dataset by weighing stage, gender, age, grade, T stage, N stage, M stage and risk score (Fig. 8A). In addition, the C-index was employed to estimate the accuracy of the nomogram, which was 0.637. Calibration curves demonstrated that nomogram functioned well in forecasting OS and actual OS at 1-, 3-, and 5-year (Fig. 8B–D).

### Relationship of ANXA10 expression with m6A modification

**Figure 9:**
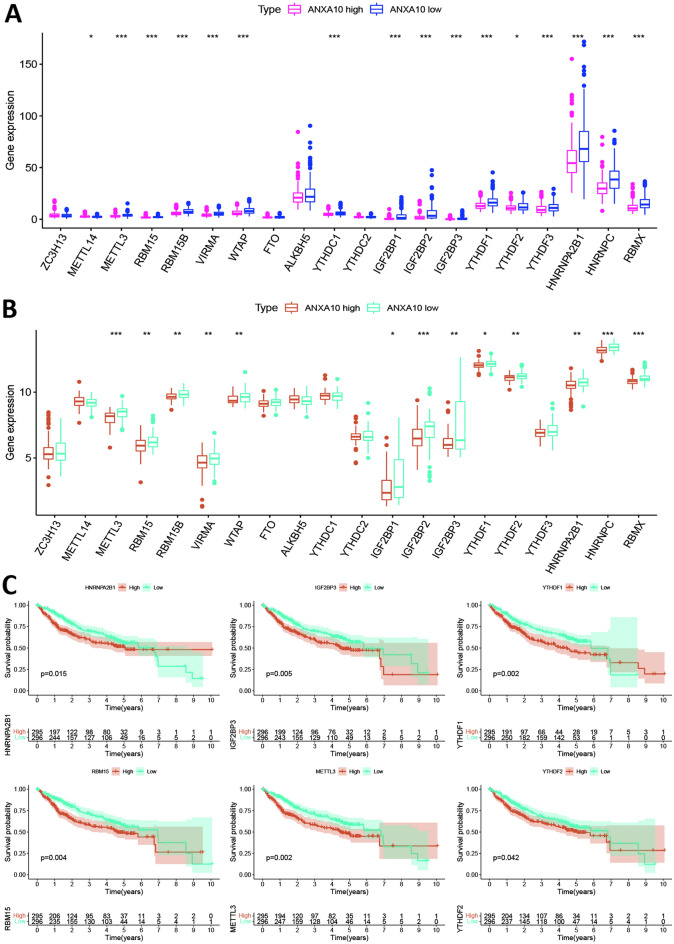
Correlation of ANXA10 expression with m6A genes in TCGA-LIHC. The m6A genes discrepancy of expression in the low and high ANXA10 expression groups with TCGA (**A**) and GSE14520 (**B**) data. (**C**) Kaplan–Meier curve of HNRNPA2B1, IGF2BP3, METTL3, RBM15, YTHDF1 and YTHDF2 in LIHC after TCGA-LIHC data and GSE14520 data were merged. *p < 0.05; **p < 0.01; ***p < 0.001.

The m6A modification plays a crucial role in the onset and progression of LIHC. To determine whether the expression of ANXA10 was associated with 20 m6A-related genes, we used the median expression value of ANXA10 to divide 374 TCGA-LIHC tumor patients into high (187 cases) and low (187 cases) expression groups. The GSE54236 data was also used to explore the difference of 20 m6A-related genes based on the expression of ANXA10. The results of Fig. 9A,B shows that the m6A genes discrepancy of expression between the low and high ANXA10 expression groups in TCGA and GSE54236 database, respectively. The intersection of Fig. 9A,B is that the expression levels of METTL3, RBM15, RBM15B, VIRMA, WTAP, IGF2BP1, IGF2BP2, IGF2BP3, YTHDF1, YTHDF2, HNRNPA2B1, HNRNPC and RBMX were decreased in the high expression group compared to those in the group with low expression. To more deeply assess the relationships between these 13 m6A-related genes and survival, TCGA-LIHC data and GSE14520 data were merged and examined using Kaplan–Meier curves. The results showed that high expression of HNRNPA2B1, IGF2BP3, METTL3, RBM15, YTHDF1 and YTHDF2 was substantially linked to a worse prognosis in LIHC (Fig. 9C). These findings suggest that ANXA10 might be closely affiliated with the m6A modification in LIHC, particularly via its regulation of HNRNPA2B1, IGF2BP3, METTL3, RBM15, YTHDF1 and YTHDF2, and ultimately affecting the progression and prognosis of LIHC.

### Construction of ANXA10 ceRNA regulatory network

**Figure 10:**
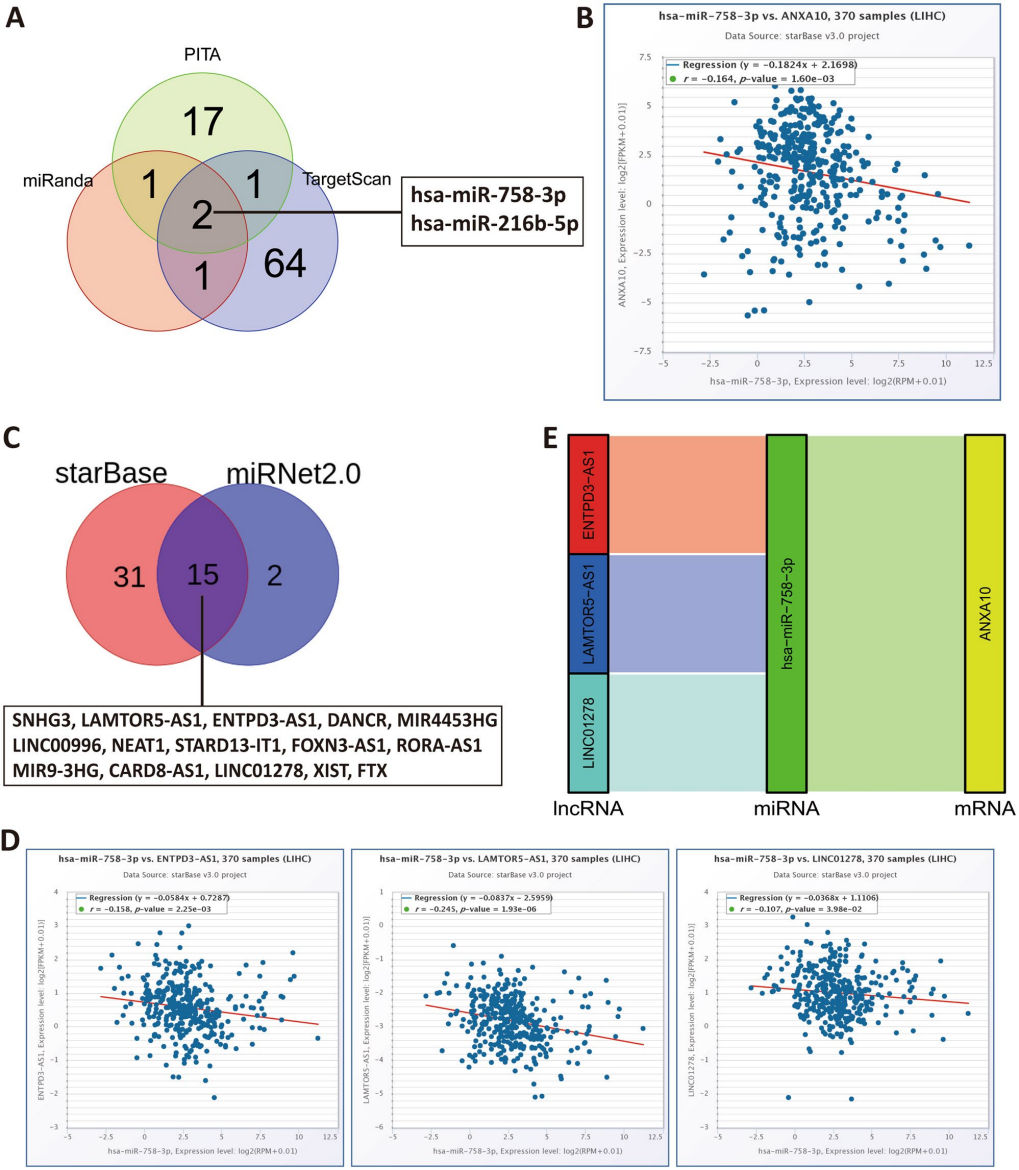
Prediction and identification of the ANXA10 ceRNA network. (**A**) Venn diagram displaying ANXA10-targeting miRNAs in the PITA, miRanda, and TargetScan tools. (**B**) Significant correlations of ANXA10 and targeted miRNAs analyzed by starBase. (**C**) Venn diagram showing the targeting-lncRNAs of hsa-miR-758-3p predicted by starBase and miRNet2.0. (**D**) Scatter plots displaying the close correlations between hsa-miR-758-3p expression and ENTPD3-AS1, LAMTOR5-AS1 and LINC01278 expression. (**E**) Sankey diagram showing the correlations in the final ceRNA network.

Previous literature reported that the ceRNA regulatory network is a useful candidate biomarker for the clinical diagnosis and therapy of hepatocellular carcinoma^32^. Therefore, we attempted to build a ceRNA network incorporating the ANXA10 gene in LIHC. The three databases miRanda, PITA, and TargetScan predicted 4, 21, and 68 ANXA10-targeting miRNAs in LIHC respectively, displaying them with a Venn diagram. The Venn diagram in Fig. 10A showed that the intersection of the three databases involved 2 targeted miRNAs (hsa-miR-758-3p and hsa-miR-216b-5p). To obtain suitable targeted miRNAs, we further analyzed the expression relationship between the 2 targeted miRNAs and ANXA10 and found that only hsa-miR-758-3p expression was inversely associated with ANXA10 expression (Fig. 10B, r = − 0.164, p = 1.60E−03).

To obtain the targeting-lncRNA of hsa-miR-758-3p in LIHC, we utilized the starBase and miRNet2.0 databases for prediction and displayed them through a Venn diagram. Figure 10C showed that the intersection of starBase and miRNet2.0 results yielded 15 lncRNAs (SNHG3, LAMTOR5-AS1, ENTPD3-AS1, DANCR, MIR4453HG, LINC00996, NEAT1, STARD13-IT1, FOXN3-AS1, RORA-AS1, MIR9-3HG, CARD8-AS1, LINC01278, XIST, and FTX). Subsequently, we used the starBase database to analyze whether these 15 lncRNAs had negative expression relationship with hsa-miR-758-3p. The results shown in Fig. 10D revealed that hsa-miR-758-3p was negatively associated with ENTPD3-AS1 (r =  − 0.158, p = 2.25E−03), LAMTOR5-AS1 (r =  − 0.245, p = 1.93E−06) and LINC01278 (r =  − 0.107, p = 3.98E−02) at the expression level. Therefore, we established 3 sets of ceRNA regulatory networks (ENTPD3-AS1—hsa-miR-758-3p—ANXA10, LAMTOR5-AS1—hsa-miR-758-3p—ANXA10 and LINC01278—hsa-miR-758-3p—ANXA10) based on the ceRNA hypothesis in Fig. 10E.

### ANXA10 overexpression inhibited cell proliferation and migration in LIHC

**Figure 11:**
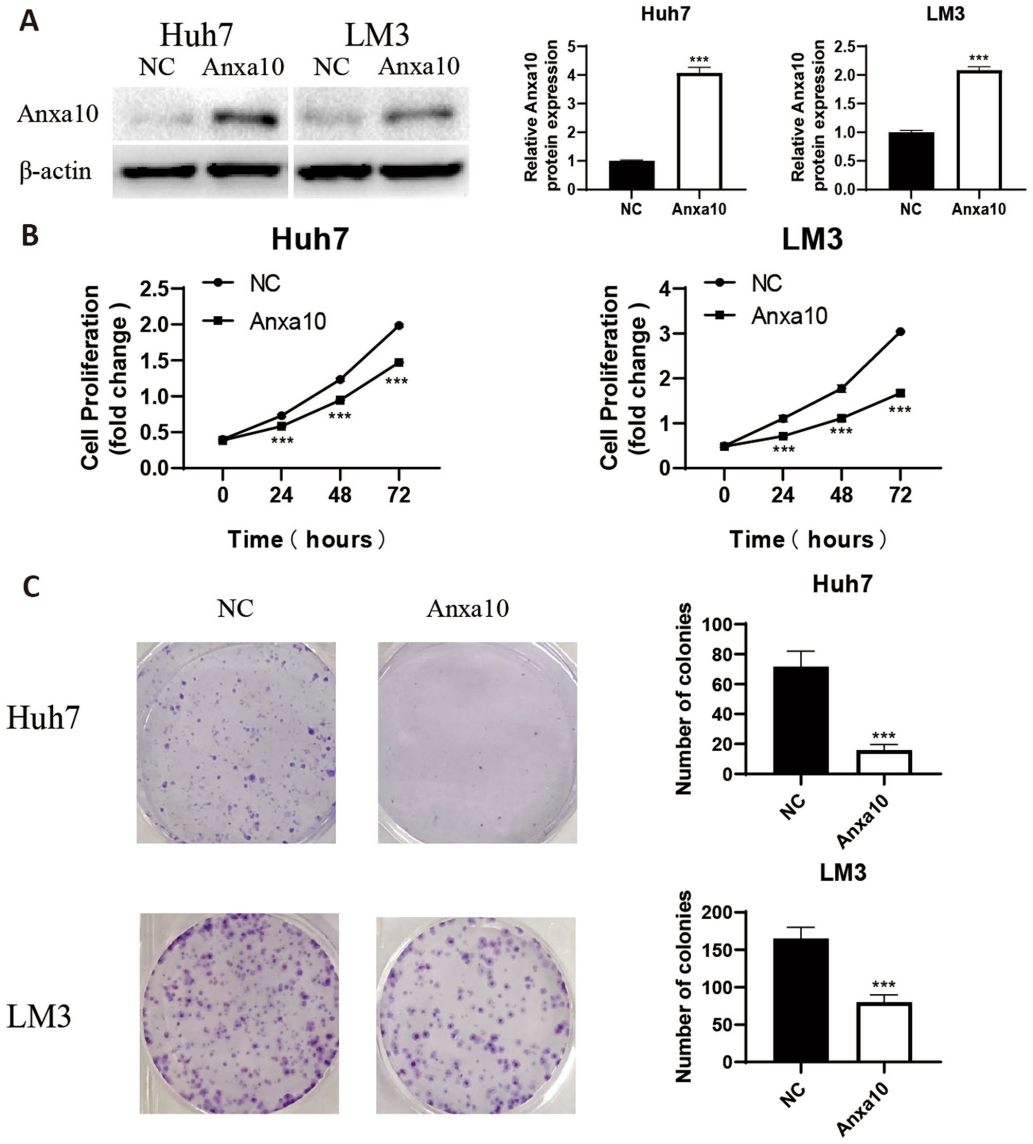

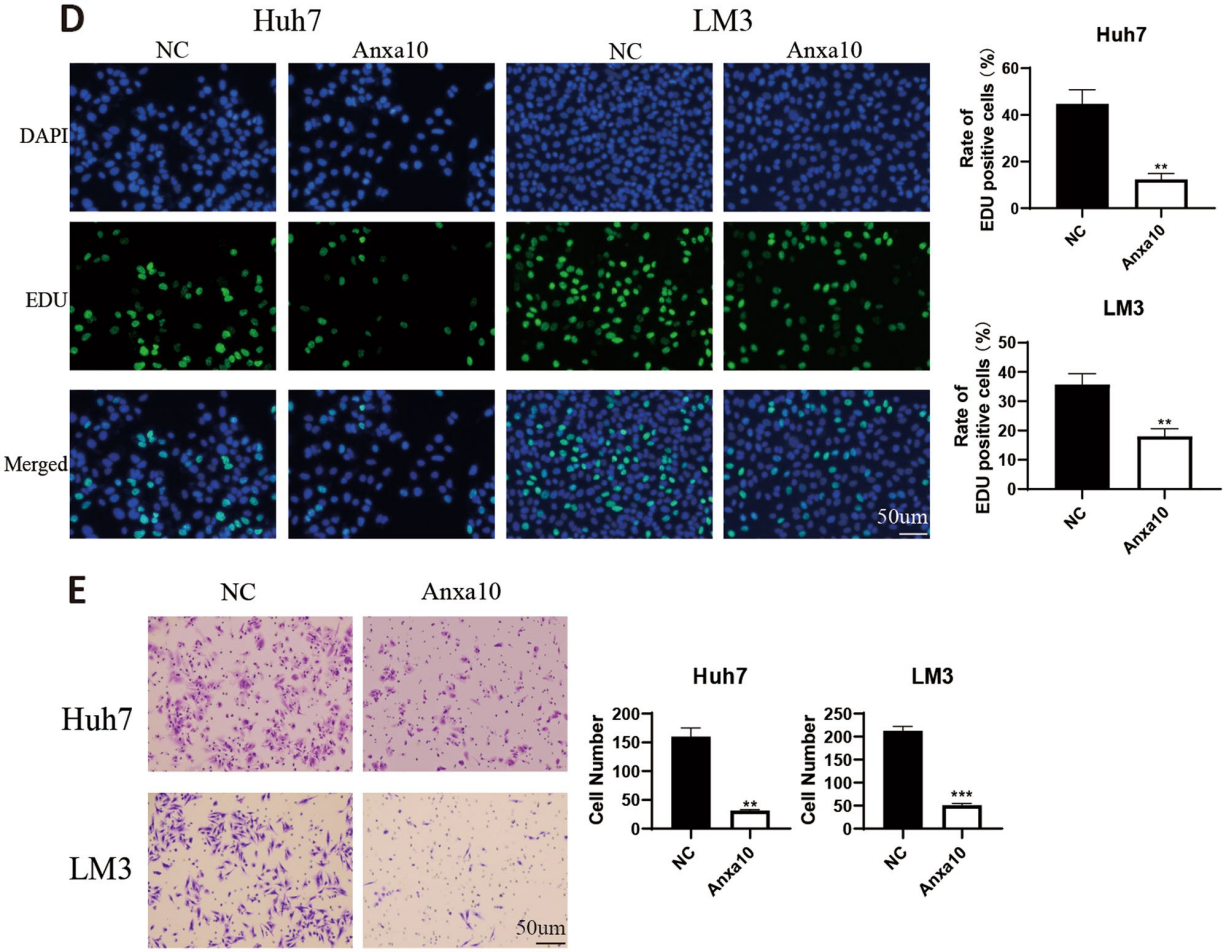
Upregulation of ANXA10 inhibited LIHC cell proliferation and migration. (**A**) The protein expression level of ANXA10 at LIHC cell lines after transfection with an ANXA10 plasmid and its negative control (NC) plasmid detected by western blotting. (**B**) MTT assay was utilized to determine the impact of ANXA10 overexpression on LIHC cell growth. (**C,D**) Colony-forming and EdU assays showed that ANXA10 overexpression restricted the proliferation. (**E**) The migration ability of LIHC cells was detected by transwell assay. *p < 0.05; **p < 0.01; ***p < 0.001.

To further identify the biological role of the ANXA10 gene in LIHC, we constructed an ANXA10 overexpression plasmid and transfected it into the Huh7 and LM3 cell lines. In Fig. 11A, the result of West-blotting trial verified that ANXA10 protein expression was significantly increased after Huh7 and LM3 cell lines were transfected. In addition, an MTT assay (Fig. 11B) revealed that the growth rates of Huh7 and LM3 cell lines were significantly decreased after ANXA10 overexpression. Next, a colony-forming assay showed that overexpression of ANXA10 caused a significant reduction in colony numbers in Huh7 and LM3 cell lines (Fig. 11C). Furthermore, the EdU assay shown in Fig. 11D indicated that the EDU positivity rate was lower in the ANXA10 upregulation group than that inthe control group. Moreover, transwell assay (Fig. 11E) showed that the number of migrated cells of Huh7 and LM3 cells was significantly reduced in the ANXA10 overexpression group. These results indicate that ANXA10 overexpression can weaken the proliferation and migration ability of LIHC cells.

## Discussion

Liver hepatocellular carcinoma is the most common cancer in the world with rising incidence. LIHC is mostly treated by surgical excision; however, because of the high relapse and migration rates, the prognosis of LIHC patients is generally dismal^33^. Owing to its occult onset, rapid progression, high relapse and migration rates, LIHC patient prognosis is poor, with a 5-year survival rate remains between 25 and 39%, and the rate of relapse in advanced-LIHC patients is approximately 80%^34^. Fortunately, the emergence of immunologic therapy and research regarding m6A modifications and the ceRNA regulatory network have provided innovative concepts for the diagnosis and therapy of malignancies. Consequently, a deeper comprehension of the occurrence and progression of LIHC will help in the identification of therapeutic targets, the innovation of more appropriate treatment approaches, and the extension of LIHC patient survival time.

Previous studies have shown that ANXA10 has both anti-tumorigenic and pro-tumorigenic mechanisms, with the dominant mechanism mainly depending on tissue specificity. Miyazawa et al. found that overexpression of ANXA10 inhibited the proliferation, migration and invasion of prostate cancer cells^35^. However, Kodaira et al. suggested that ANXA10 interacts with tumor-associated macrophages to promote esophageal carcinogenesis^36^. In the present research, we identified significant differences in ANXA10 expression among LIHC patients. To further examine the potential role of ANXA10 in LIHC, we performed enrichment analysis of coexpressed genes, found the correlations between genes and immunity, and constructed a prognostic model. We also explored the impact of ANXA10 on m6A modification and the ceRNA network in LIHC.

The TIMER tool showed that ANXA10 to be expressed at low levels in 2 cancers, namely, CHOL and LIHC. The results of TCGA-LIHC data, GSE14520 data and GEPIA2 showed ANXA10 expression to also be meaningfully low in tumor tissues. In vitro experiments confirmed that ANXA10 was expressed at low levels in LIHC cell lines in both mRNA and protein levels. This expression difference was also observed in previous studies^11^. Furthermore, we found that LIHC patients with high ANXA10 expression had favorable survival. Moreover, the results of TISIDB and GEPIA2 showed that patients with high ANXA10 expression had lower pathological stage and grade. Further analysis of in vitro experiments confirmed that overexpression of ANXA10 inhibited the proliferation and migration of LIHC cells. These findings indicate that ANXA10 may act as a tumor suppressor in LIHC. In previous study, down-regulation of ANXA10 has been proved to be associated with p53 mutation^11^. Indeed, the functions of mutant p53 could contribute to cancer proliferation and metastasis^37^. In recent study, Cao et al. found that the membrane‐associated RING‐CH (MARCH) ligases played an important role in LIHC and ANXA10 belonged to one of MARCH ligase-related genes^38^. To our knowledge, MARCH ligases belong to E3 ubiquitin ligases, which can determine the specificity of protein substrates and is considered as a potential diagnostic and therapeutic target for cancer^39^. Additionally, MARCH ligases control the function of important immunoreceptors, including histocompatibility complex class (MHC) molecules and the costimulatory molecule CD86^38^. It is widely known that MHC is related to cancer immune evasion^40^. Moreover, CD86 provides similar costimulatory signals for T cell proliferation, cytokine production, and generation of cytotoxic lymphocyte^41^. Therefore, we speculate that ANXA10 plays an anti-cancer role in LIHC, which may be related to p53 mutation, E3 ubiquitin ligases and immunoreceptors. In conclusion, ANXA10 has the potential to function as a prognostic, diagnostic and therapeutic target in LIHC.

To more deeply comprehend ANXA10’s function and mechanism, we utilized LinkedOmics to scrutinize ANXA10 coexpressed genes in LIHC. KEGG pathway analysis showed that ANXA10 was positively associated with Fatty acid degradation and inversely associated with Hippo signaling pathway, Wnt signaling pathway, Hedgehog signaling pathway and Cell cycle. It has been suggested that cancer cell proliferation can be suppressed by enhancing fatty acid degradation^42^. Yang et al. found that stimulation of the Hippo signaling pathway promotes the HCC cell growth and tumorigenesis^43^. Huang et al. suggested that stimulation of the Wnt signaling pathway promotes the progression of hepatoma cells^44^. Shi et al. demonstrated that stimulation of the Hedgehog signaling pathway accelerates the growth of HCC cells^45^. Past research proved that some suppressors exert effective anticancer effects by inhibiting the cell cycle^46^. These findings indicate that ANXA10 can act as a tumor inhibitor through multiple biological pathways in LIHC.

To explore the relationship between ANXA10 and immunity in LIHC, we conducted inclusive analysis using a variety of methods. TIMER database analysis found that ANXA10 SCNA was positively correlated with the abundances of B cell and Dendritic cell. Additionally, the ssGSEA results indicated that the levels of Cytotoxic cells, DC, Neutrophils and Th17 cells increased in the ANXA10 high expression group, while the levels of Macrophages and NK CD56bright cells decreased. For further analysis, CIBERSORTx analysis showed that the abundance of M1 macrophages was positively correlated with the expression of ANXA10. Dendritic cells^47^ and M1 macrophages^48^ have been shown to have antitumor effects. It was reported that B cells^49^ and Cytotoxic cells^50^ in tumors are associated with a good prognosis. In contrast, NK CD56bright cells are reported to be inversely associated with survival^51^. Additionally, KEGG enrichment analyses of ANXA10-related immunoregulators showed that Cytokine-cytokine receptor interaction, Toll-like receptor signaling pathway, Cell adhesion molecules and Th17 cell differentiation were related to ANXA10-mediated immune events. These results suggest an important immunomodulatory role for ANXA10 in LIHC.

To explore the potential prognostic role of ANXA10-related immunomodulators, we attempted to establish a gene prognostic signature. Qi et al. analyzed ferroptosis-related genes and constructed a 9-gene signature, which had good prognostic accuracy for predicting colon cancer^52^. In the present research, we constructed a 3-gene prognostic signature for LIHC based on ANXA10-related immune regulators. The risk score generated from the immune gene signature exhibited a meaningful relationship with survival in TCGA-LIHC cohort patients. By multivariate and univariate Cox regression analyses, the risk score was determined to be an independent predictor in LIHC. Then, we combined signature and clinical features to construct a nomogram for individualized prognostic forecasting with a C-index of 0.637. These results provide a quick and precise way to predict the prognosis of LIHC patients in the clinic.

The m6A methylation modification has reported to be related to tumor proliferation, angiogenesis, metastasis, immunity, and other processes^53^. To determine whether ANXA10 was associated with m6A in LIHC, we performed m6A methylation modification analysis using TCGA-LIHC data and GEO data. In the present study, our results showed that the expression extents of METTL3, RBM15, RBM15B, VIRMA, WTAP, IGF2BP1, IGF2BP2, IGF2BP3, YTHDF1, YTHDF2, HNRNPA2B1, HNRNPC and RBMX were statistically decreased in the high ANXA10 expression group. In addition, Kaplan–Meier curve analysis showed that patients with high expression of HNRNPA2B1, IGF2BP3, METTL3, RBM15, YTHDF1 and YTHDF2 had worse survival. Luo et al. found that knockdown of HNRNPA2B1 impaired the proliferation and invasion of hepatoma cells^54^. Zhang et al. discovered that hsa-circ-0026134 promoted the proliferation and invasion of IGF2BP3-mediated LIHC cells by sponging miR-127-5p^55^. Wang et al. demonstrated that silencing METTL3 could impair the proliferation, migration and invasion of LIHC cells^56^. Cai et al. found that RBM15-mediated m6A modification promoted HCC progression through the IGF2BP1-YES1-MAPK axis^57^. Luo et al. found that YTHDF1 promoted LIHC cell proliferation by activating the PI3K/AKT/mTOR signaling pathway and promoted LIHC cell migration by driving epithelial-mesenchymal transition (EMT)^58^. Zhang et al. demonstrated that YTHDF2 could promote the liver cancer stem cell phenotype and cancer metastasis^59^. These outcomes suggest that the tumor suppressor effect of ANXA10 is related to m6A; ANXA10 may regulate HNRNPA2B1, IGF2BP3, METTL3, RBM15, YTHDF1 and YTHDF2 to affect the methylation level of LIHC (Supplementary Information).

The ceRNA networks connect the functions of mRNAs that code for proteins to those of noncoding RNAs comprising miRNAs, lncRNAs, pseudogene RNAs, and circular RNAs^60^. The etiology of numerous prevalent malignancies is associated with competing endogenous RNAs (ceRNAs), such as endometrial cancer, liver cancer, lung cancer, breast cancer, gastric cancer and prostate cancer^61^. Sun et al. found that circ-0000105 indirectly increased PIK3R1 expression by binding miR-498, thereby promoting the proliferation of hepatoma cells^62^. In our study, results from three databases revealed that ANXA10 has 2 potential upstream miRNAs, but only hsa-miR-758-3p was inversely correlated with ANXA10 expression. Zhou et al. found that ovarian cancer patients with high expression of hsa-miR-758-3p had a worse prognosis^63^. Then, we further forecasted the upstream lncRNAs of hsa-miR-758-3p through the starBase and miRNet2.0 databases. Correlation analysis showed that only the expression levels of ENTPD3-AS1, LAMTOR5-AS1 and LINC01278 were negatively correlated with those of hsa-miR-758-3p. Functional experiments showed that overexpression of ENTPD3-AS1 inhibited cell proliferation in renal cancer cell lines^64^. Pu et al. found that LAMTOR5-AS1 could markedly impair the proliferation of osteosarcoma cells^65^. Lin et al. found that Linc01278 inhibited the cell proliferation of papillary thyroid cancer by impelling apoptosis and mitigated the effects of cancer cells migration and invasion by regulating the EMT process^66^. These studies all suggest that the ceRNA network of ANXA10 may play an important role in LIHC, but further experimental confirmations are needed.

In conclusion, this is the first multidimensional analysis of the potential biological functions of ANXA10 in LIHC. ANXA10 may inhibit the proliferation and migration of LIHC cells by activating or inhibiting multiple signaling pathways, regulating the infiltration of immune cells, affecting the methylation level of tumors, and participating in the regulation of ceRNA networks. Therefore, ANXA10 may be a meaningful biomarker for LIHC diagnosis, treatment and prognosis prediction.

### Ethics approval

This study was approved by the Ethics Committee of the Second Affiliated Hospital of Chongqing Medical University and acquired informed consent from all patients who participated in this study. All methods were performed in accordance with the Helsinki declaration guidelines and regulations.

## Supplementary Information

Supplementary Information.

## Data Availability

The datasets analyzed during the current study are available in the repository TCGA website (https://portal.gdc.cancer.gov/).
