# Supplementary figures and images for: ANXA10 is a prognostic biomarker and suppressor of hepatocellular carcinoma: a bioinformatics analysis and experimental validation
Source: Sci Rep. 2023 Jan 28;13:1583. doi: 10.1038/s41598-023-28527-x (PMC9884230; doi:10.1038/s41598-023-28527-x)

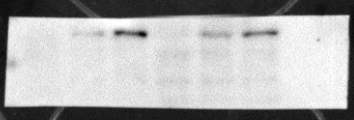

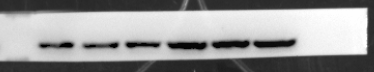


ANXA10

β-actin

Supplement: Supplementary file 1 — Supplementary Information. [file 41598_2023_28527_MOESM1_ESM.docx]
